# Antimicrobial peptide CEC-TY1: a potential antibacterial drug against carbapenem-resistant *Klebsiella pneumoniae*

**DOI:** 10.1128/spectrum.00543-25

**Published:** 2025-10-16

**Authors:** Yaozhu Yang, Hailong Yang, Qiuyue He, Min Niu, Kai Yang, Yongshi Zhao, Shumin Liu, Yan Du

**Affiliations:** 1 Department of Clinical Laboratory, the First Affiliated Hospital of Kunming Medical University 36657, Kunming, China; 2 Yunnan Key Laboratory of Laboratory Medicine, Kunming, China; 3 Yunnan Province Clinical Research Center for Laboratory Medicine, Kunming, China; 4 School of Basic Medical Sciences, Kunming Medical University 71240, Kunming, Yunnan, China; University of Torino, Turin, Italy

**Keywords:** *Klebsiella pneumoniae*, carbapenem resistance, antimicrobial peptides, infection, antibacterial activity

## Abstract

Carbapenem-resistant *Klebsiella pneumoniae* (CRKP) infections in hospitals and the issues arising from their rapid spread have become significant public health challenges. Therefore, a new class of effective and safe antibacterial drugs is urgently required. In this study, we identified an antimicrobial peptide in the cecropin family, cecropin-TY1 (CEC-TY1), from the salivary glands of the horsefly. CEC-TY1 showed significant antibacterial activity against gram-negative bacteria, at concentrations up to 100 μg/mL, with no observed cytotoxicity or hemolytic activity against cells. Moreover, CEC-TY1 protected mice from lethal infections of CRKP *in vivo*, improving the survival rate of the infected mice. This peptide also alleviated excessive and harmful inflammatory responses by inhibiting the production of interleukin (IL)-6, tumor necrosis factor-α, and IL-1β.

**IMPORTANCE:**

Antimicrobial peptides are a promising alternative to antibiotics in the current antibiotic resistance crisis. Specifically, the antimicrobial peptide cecropin-TY1 (CEC-TY1), identified in the salivary glands of horseflies, showed significant antibacterial activity against carbapenem-resistant *Klebsiella pneumoniae* (CRKP), both *in vitro* and *in vivo*. CEC-TY1 also has selective toxicity and low hemolytic activity and is therefore a potential therapeutic agent for acute CRKP infection.

## INTRODUCTION

*Klebsiella pneumoniae* is one of the most common opportunistic gram-negative pathogens in clinical settings and is also one of the main bacteria responsible for abdominal infections in individuals with compromised immune systems (1–3). The discovery of antibiotics marked a significant revolution in human healthcare. However, although antibiotics are the most advanced therapeutic agents in human history, their misuse has reduced their effectiveness in treating infections, and bacterial antibiotic resistance has once again put human health at risk (4, 5). Bacteria of the carbapenem-resistant *Enterobacteriaceae* pose a global public health threat and are associated with infections with high mortality rates because there are limited options for their treatment. The prevalence of carbapenem-resistant *Klebsiella pneumoniae* (CRKP) is rapidly increasing and presents a significant challenge to global health.

Intra-abdominal infections (IAIs) are primarily caused by the invasion of the abdominal cavity by pathogenic bacteria. The host’s immune system triggers an inflammatory response, accompanied by the exudation of inflammatory cells. Lipopolysaccharide is an important component of the outer membrane of the cell wall of gram-negative bacteria. It can promote the expression and release of cytokines such as interleukin-6 (IL-6), IL-1β, and tumor necrosis factor-α (TNF-α), and therefore is an important inducer of inflammatory injury in the body (6). Severe inflammation can lead to multiple-organ failure, septic shock, and even death (7–11), so the damage caused is extremely serious. According to the relevant literature, gram-negative bacteria are the primary causes of IAIs, with a detection rate as high as 75.36%. The most common pathogenic bacteria of IAIs are *K. pneumoniae, Escherichia coli*, and other bacteria (9). Currently, the treatment of IAIs relies primarily on antibiotic therapy (12, 13). However, infections caused by CRKP typically lack effective treatment options and are associated with high mortality rates (14, 15).

The development of antibiotics is currently at a bottleneck, and new types of antibiotics or antibiotic alternatives for the treatment of infections induced by CRKP are urgently required (16). Antimicrobial peptides (AMPs) are an important part of the innate defense system, with a wide range of activities, including the ability to directly kill bacteria, fungi, and even cancer cells (17–20), and they have great therapeutic potential against antibiotic-resistant bacteria (21–25).

The salivary glands of blood-sucking insects are rich in a variety of anticoagulant, anti-inflammatory, antibacterial, and immunosuppressive substances (26–28), including cecropin-TY1 (CEC-TY1). In this study, we used an *in vitro* drug sensitivity test and a bactericidal kinetics test to determine whether CEC-TY1 has antibacterial activity *in vitro*. We then evaluated the safety of CEC-TY1 through cell-level verification and investigated the possible antibacterial mechanism of CEC-TY1. Finally, we established a mouse model of acute CRKP peritoneal infection to observe the antibacterial effect of CEC-TY1 *in vivo*. Our research has advanced the understanding of the anti-infective capabilities of antimicrobial peptides and offered a promising antibiotic alternative for the treatment of CRKP infections.

## MATERIALS AND METHODS

### Antimicrobial peptides and antibiotic

The antimicrobial peptide used in the experiment, CEC-TY1 (GWLKKIGKKIERVGQNVRNAAISTLPIAQGAAGVAGALN-NH_2_), was synthesized and provided by Shanghai Jietai Biotechnology Co., Ltd., with a final purity >95%.

Antibiotic: polymyxin B (PB) was purchased from Coolaber Technology Co. (Beijing, China).

### Bacterial strains

Carbapenem-resistant *Klebsiella pneumoniae* (CRKP_1, CRKP_5, CRKP_9, CRKP_11, CRKP_13) strains were isolated from samples sent for testing from various departments of the First Affiliated Hospital of Kunming Medical University. The standard strains *Staphylococcus aureus* ATCC 29213, *Enterococcus faecalis* ATCC 29212, *E. coli* ATCC 25922, *Pseudomonas aeruginosa* ATCC 27853, and *K. pneumoniae* ATCC BAA-1706 are preserved in this laboratory, with all experimental strains stored in glycerol at −80°C.

### Cell line

The mouse mononuclear macrophage leukemia cell RAW 264.7 was purchased from Shanghai Saibikang Biotechnology Co., Ltd. Cell line integrity was confirmed using short tandem repeat analysis. Mouse peritoneal macrophages (MPMs) were derived from primary cells obtained from the mouse peritoneal cavity. The isolation and culture of MPMs were performed following previous methods with minor modifications (29).

### Experimental animals

Specific pathogen free (SPF)-grade female kunming (KM) mice (6 to 8 weeks old, 20 g–25 g) were purchased from the Experimental Animal Center of Kunming Medical University.

### Bacterial culture

The bacterial preservation tubes used in the experiment were placed on ice, and samples were inoculated onto fresh Columbia blood agar plates with marked quadrants, followed by incubation at 37°C for 16–18 h. A single bacterial colony was then inoculated into Luria-Bertani (LB) liquid medium and cultured under shaking conditions at 37°C and 200 r/min until it reached the logarithmic growth phase.

### Antibacterial activity

The antibacterial activity was detected using the micro-broth dilution method, which was conducted in accordance with previously established protocols, with minor modifications (30). A 0.5 McFarland turbidity standard suspension (approximately 1 × 10^8^ colony-forming unit [CFU]/mL) was prepared from a single colony of fresh strains obtained after recovery. The prepared bacterial suspension was then diluted with Mueller-Hinton (M-H) broth to a concentration of 1 × 10^6^ CFU/mL for later use.

In a 96-well plate, CEC-TY1 and PB were serially diluted and mixed with the bacterial suspension (100 µL/well) to achieve final concentrations ranging from 64 to 0.125 µg/mL. The mixture was incubated at 37°C for a total of 16–18 h, and the optical density at 600 nm was measured using a microplate reader. The concentrations corresponding to wells with no significant bacterial growth were determined to be the minimum inhibitory concentrations (MICs) of CEC-TY1 and PB. Each experiment was independently repeated three times. For the minimum bactericidal concentration (MBC), from the previous experiment with CEC-TY1, 10 µL of bacterial suspension greater than the MIC was taken from each well of the 96-well plate and inoculated onto sterile LB agar plates. The plates were incubated at 37°C for 16 h. The minimum concentration at which no sterile colonies grew was defined as the MBC.

### Bactericidal kinetics experiment

The change in the number of viable bacteria after treatment was assessed to evaluate the bactericidal kinetics of CEC-TY1. As previously described, *Klebsiella pneumoniae* ATCC BAA-1706, CRKP_1, CRKP_5, CRKP_9, CRKP_11, and CRKP_13 were cultured to the logarithmic growth phase, diluted to 1 × 10^5^ CFU/mL, and incubated with CEC-TY1 at a concentration of 4 × MIC and PB at 37°C with shaking at 200 r/min. Samples of 10 µL were taken at 0, 1, 2, 4, 6, 8, and 12 h, diluted in freshly prepared sterile LB broth (with different dilution factors for each group), and 20 µL of each diluted sample was plated on LB agar plates. The plates were incubated overnight at 37°C, and the number of viable bacterial colonies was counted the following day. Sterile phosphate buffer solution (PBS) and 4 × MIC PB served as negative and positive controls, respectively.

### Propidium iodide (PI) fluorescent staining experiment

The concentration of the resuspended bacterial suspension was adjusted to 2 × 10^8^ CFU/mL. Different volumes of CEC-TY1 were added to achieve final concentrations of 1 × MIC, 2 × MIC, and 4 × MIC, with the blank group receiving no CEC-TY1. The mixture was thoroughly mixed and incubated at 37°C for 1.5 h. After incubation, an equal volume of PI staining solution was added to achieve a final concentration of 100 µg/mL, and the samples were incubated in the dark for 15 min. The mixture was centrifuged at 3,000 rpm for 10 min to remove the supernatant, washed three times with sterile PBS, and centrifuged again to remove excess dye. The bacterial pellet was resuspended in 1 mL of sterile PBS, and red fluorescence was observed under an inverted fluorescence microscope, capturing images. The above steps were repeated, and the resuspended bacterial solution was added to a sterile 96-well plate. Fluorescence intensity was measured using a multifunctional microplate reader, with an excitation wavelength of 535 nm and an emission wavelength of 615 nm.

### SEM

A bacterial suspension with a concentration of 1 × 10^8^ CFU/mL was prepared and then added to CEC-TY1 M-H broth culture medium to achieve a final concentration of 2 × MIC. The control group was supplemented with MH broth culture medium without CEC-TY1. The cultures were incubated at 37°C for 16 h. The bacterial pellet was collected by centrifugation, the culture medium was discarded, and the pellet was washed three times with PBS. The suspension was centrifuged (8,000 rpm, 5 min), the PBS was discarded, and fixative for electron microscopy was added. The bacteria were mixed and fixed at room temperature in the dark for 30 min, then transferred to 4°C for storage. The samples were rinsed three times with 0.1 M phosphate buffer (pH = 7.4), each for 15 min. They were then fixed with 1% osmium tetroxide at room temperature in the dark for 1–2 h, then rinsed three times with 0.1 M phosphate buffer (pH = 7.4), each for 15 min. Sequentially, 30%, 50%, 70%, 80%, 90%, 95%, 100%, and 100% ethanol were added, each for 15 min, followed by isopropyl acetate for 15 min. After drying, the samples were coated with gold and photographed.

### Median lethal dose (LD_50_)

The LD_0_ and LD_100_ of *K. pneumoniae* ATCC BAA-1706 and CRKP_9 were screened in the preliminary experiment.

Sixty SPF-grade female KM mice were randomly divided into 10 groups, with six mice in each group. Groups 1–5 were designated as the *K. pneumoniae* ATCC BAA-1706 experimental groups, while groups 6–10 were designated as the CRKP_9 experimental groups. After a 2 day acclimatization period in the new environment, the experiments commenced. Based on preliminary experimental results, the concentrations of the bacterial solution injected into groups 1–5 were 9.5 × 10^8^, 6 × 10^8^, 4. × 10^8^, 2 × 10^8^, and 1 × 10^8^ CFU/mL, respectively. The concentrations for groups 6–10 were 6 × 10^8^, 3 × 10^8^, 1 × 10^8^, 9 × 10^7^, and 7.5 × 10^7^ CFU/mL, respectively. The mice were infected via intraperitoneal injection, and after injection, they were provided with sterilized mouse feed and drinking water. The survival status of the mice was observed continuously for 7 days, with records taken every 24 h. The LD_50_ values for the *K. pneumoniae* ATCC BAA-1706 and CRKP_9 strains were calculated using the Bliss method.

### Cytotoxicity test

The cytotoxic activity of CEC-TY1 on MPMs and RAW 264.7 cells was determined using reported experimental methods (30, 31). The cytotoxic activity of CEC-TY1 on MPMs and RAW 264.7 cells was dvaluated. A total of 1 × 10^4^ cells (100 µL/well) were added to a 96-well plate and incubated with CEC-TY1 (10, 25, 50, and 100 µg/mL) in a cell culture incubator (37°C, 5% CO_2_) for 24 h. After incubation, 10 µL of cell counting kit-8 (Biosharp, China) solution was added to each well, and after an additional 2 h of incubation, the absorbance was measured at 450 nm.

### Hemolytic activity test

The hemolytic activity test was used to verify the effect of CEC-TY1 on red blood cells, and certain changes were made according to the experimental methods reported previously (32). Experiments were conducted using red blood cells from healthy adults and mice. A total of 400 µL of peripheral blood from healthy adults or mice was slowly added to 10 mL of sterile PBS, gently mixed by inverting the tube until homogeneous (1,000 × *g*, 10 min). The supernatant was carefully removed, and the precipitated red blood cells were washed two to three times with sterile PBS, centrifuging until the supernatant was clear and transparent. The red blood cells were then prepared into a 1% (vol/vol) suspension using sterile PBS, with 100 µL of the red blood cell suspension added to each well, followed by the addition of 100 µL of CEC-TY1, resulting in final concentrations of 10, 25, 50, and 100 µg/mL for CEC-TY1 in the wells. For the negative control, 100 µL of sterile PBS was added, while the positive control received 1% Triton X-100. Each group included three replicates, and each experiment was independently repeated three times. The plates were incubated in a culture chamber at 37°C for 1.5 h, then the liquid was transferred from the 96-well plate to a 1.5 mL centrifuge tube (1,000 × *g*, 10 min). Subsequently, 100 µL of the supernatant from each tube was transferred to a new sterile 96-well plate, and the absorbance was measured at 540 nm.

### Mouse abdominal infection model

According to the results of median lethal dose, the bacterial solution concentrations used in subsequent animal experiments were *K. pneumoniae* ATCC BAA-1706 (3.5 × 10^8^ CFU/mL) and CRKP_9 (0.9 × 10^8^ CFU/mL), respectively. *K. pneumoniae* ATCC BAA-1706 and CRKP_9 were injected intraperitoneally. After 2 h of bacterial injection, PBS was injected intraperitoneally in the model group, CEC-TY1 (10 mg/kg) was injected intraperitoneally in the experimental group, and PB (10 mg/kg) was injected into the positive control group. After 24 h, sterile mouse blood and peritoneal lavage fluid were collected to detect the levels of inflammatory factors (TNF-α, IL-6, IL-1β) using the enzyme-linked immunosorbent assay method. To assess bacterial colonization in the mice, the lungs, liver, kidneys, and spleen were collected, weighed, and then ground with 1 mL of sterile PBS. Subsequently, the organ homogenate and peritoneal lavage fluid were diluted with PBS (using different dilution factors for different groups and organs) and inoculated onto LB agar plates to count the number of viable bacteria.

### Survival experiment

In order to further evaluate the therapeutic effect of CEC-TY1 on bacterial infection, 48 SPF-grade KM mice were randomly divided into eight groups, with six mice in each group. The experiment began after 2 days of adaptation to the new environment. The blank control group was injected with 0.5 mL of PBS, while the model group received injections of *K. pneumoniae* ATCC BAA-1706 or CRKP_9 at concentrations of 3.5 × 10^8^ CFU/mL and 0.9 × 10^8^ CFU/mL, respectively, in equal volumes (0.5 mL) via intraperitoneal injection to infect the mice. The experimental group was administered CEC-TY1 (10 mg/kg) intraperitoneally 2 h after the bacterial injection, and the positive control group was given PB. The injected mice were provided with normal sterilized rodent chow and drinking water, and their survival was continuously monitored for 7 days, with observations recorded every 24 h.

### Statistical analysis

SPSS 24.0 statistical software was used for data analysis. All the data were expressed as the mean ± standard deviation of at least three independent experiments. The Bliss method was used to calculate the lethal dose of bacteria. The data were statistically analyzed and graphed using GraphPad Prism 8, and the statistical differences were analyzed by one-way analysis of variance and paired *t*-test on log-transformed data. *P* < 0.05, *P* < 0.01, and *P* < 0.001 were considered statistically significant.

## RESULTS

### Antibacterial activity of CEC-TY1 against bacteria

The antibacterial activity of CEC-TY1 against gram-positive bacteria such as *S. aureus* and *E. faecalis* is relatively weak, with a MIC >64 µg/mL, indicating that the inhibitory effect of CEC-TY1 on gram-positive bacteria is limited. However, for the gram-negative bacteria used in the experiment, the experimental data showed that their MIC values ranged from 2 to 4 μg/mL and MBC values ranged from 2 to 8 μg/mL. This result indicates that CEC-TY1 has significant inhibitory ability against gram-negative bacteria, and its antibacterial activity remains significant when facing CRKP strains, verifying the good antibacterial effect of CEC-TY1 on CRKP strains.

### Bactericidal kinetic curve of CEC-TY1

**Fig 1:**
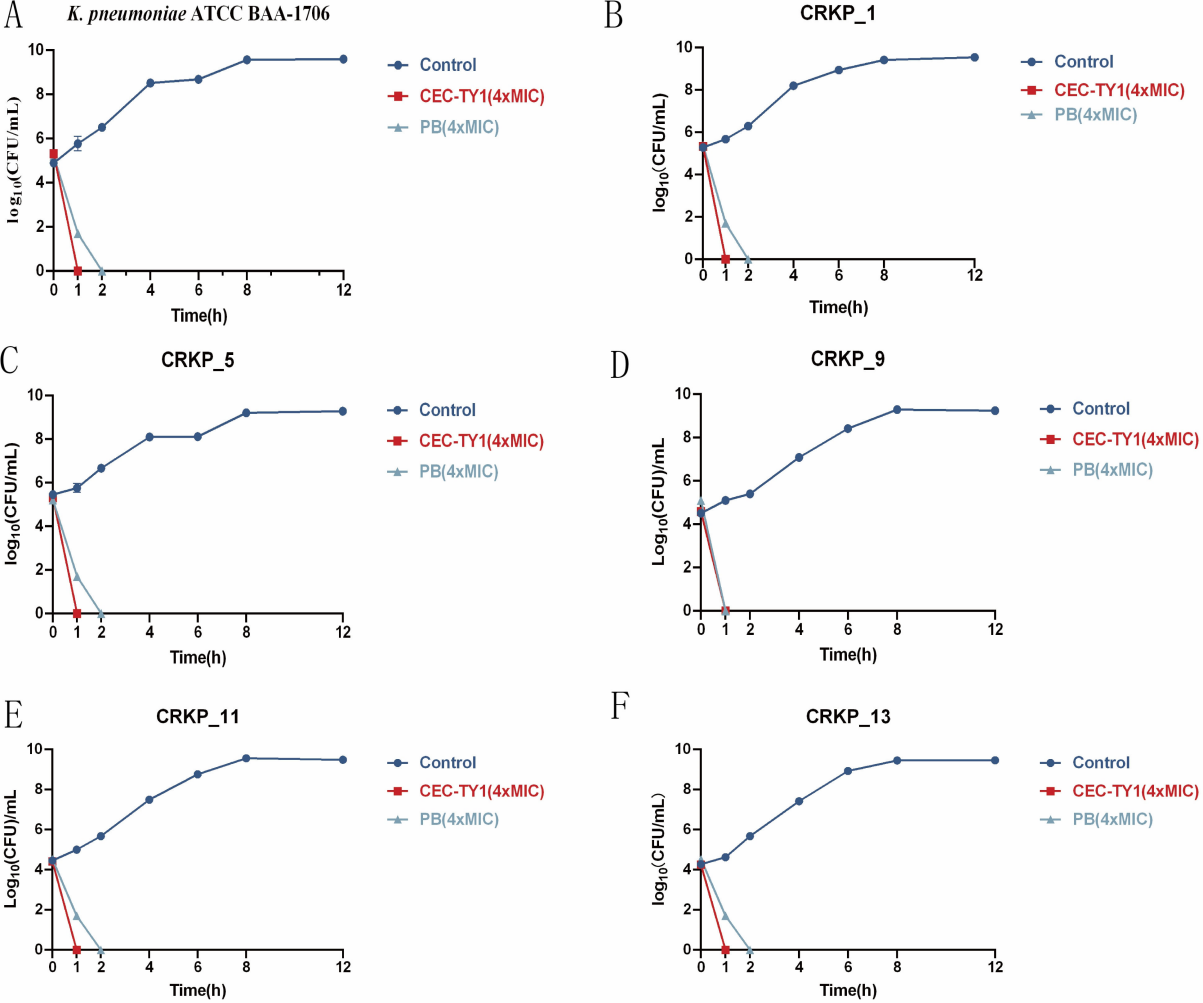
Temporal bactericidal kinetics of CEC-TY1. (**A**) *K. pneumoniae* ATCC BAA-1706, (**B**) CRKP_1, (**C**) CRKP_5, (**D**) CRKP_9, (**E**) CRKP_11, and (**F**) CRKP_13, each bacterial fluid concentration of 1 × 10^5^ CFU/mL, and 4 × MIC treatments for 0, 1, 2, 4, 6, 8, and 12 h. PB is a positive control. The bacteriological kinetics of CEC-TY1 are shown in the figure.

To further confirm the bactericidal effect of CEC-TY1, we investigated the bactericidal kinetics of CEC-TY1 against CRKP. The experimental results are shown in Fig. 1, where CEC-TY1 at 4 × MIC exhibited a rapid bactericidal ability against all strains used in the experiment, killing all bacteria within 1 h. This discovery not only confirmed that CEC-TY1 has strong antibacterial activity against CRKP, but also indicated that the rapid killing effect of CEC-TY1 on carbapenem-resistant enterobacterales strains was comparable to that of the polymyxin B control.

### PI fluorescent dye staining results

In order to investigate the antibacterial mechanism of CEC-TY1, *K. pneumoniae* ATCC BAA-1706 and CRKP_9 were stained with PI fluorescent dye solution, observed using an inverted fluorescence microscope, and fluorescence intensity was detected by fluorescence microplate reader. When the bacterial cell is damaged, PI can pass through the damaged bacterial cell membrane and bind with the bacterial DNA to emit red fluorescence, but the PI dye cannot penetrate the undamaged bacterial cell membrane; thus, it can be used to detect the integrity of the bacterial cell membrane (33).

**Fig 2:**
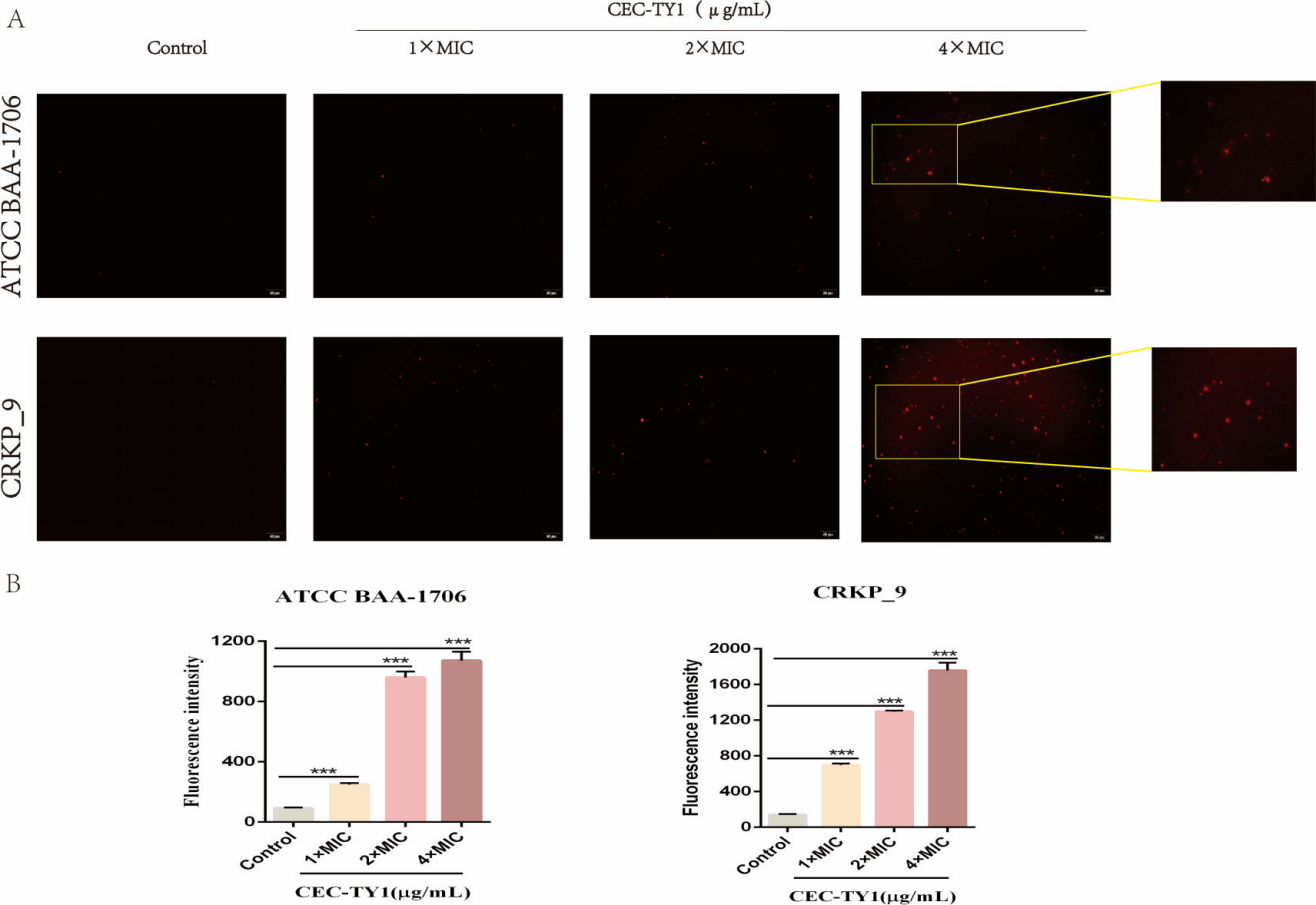
Effect of CEC-TY1 on the cell membrane of *K. pneumoniae* ATCC BAA-1706 and CRKP_9 bacteria. (**A**) The results of PI staining were observed by inverted fluorescence microscope (scale bar = 50 µm), and the fluorescence contrast in Fig. 2A was enhanced using Photoshop software. (**B**) Fluorescence intensity was measured with enzyme-labeled instrument microplate reader. *P < 0.05, **P < 0.01, and ***P < 0.001.

PI staining results of the untreated negative control group and the experimental group (1 × MIC, 2 × MIC, 4 × MIC CEC-TY1 groups) are shown in Fig. 2A. The bacteria with red fluorescence in the experimental group were significantly more than those in the control group, especially in the 4 × MIC CEC-TY1 group. The permeability of bacterial cell membranes in the experimental group was significantly higher than that in the control group. A concentration-dependent increase in PI fluorescence intensity was observed with CEC-TY1 treated bacteria, reflecting increased membrane permeability, as shown in Fig. 2B.

### SEM

**Fig 3:**
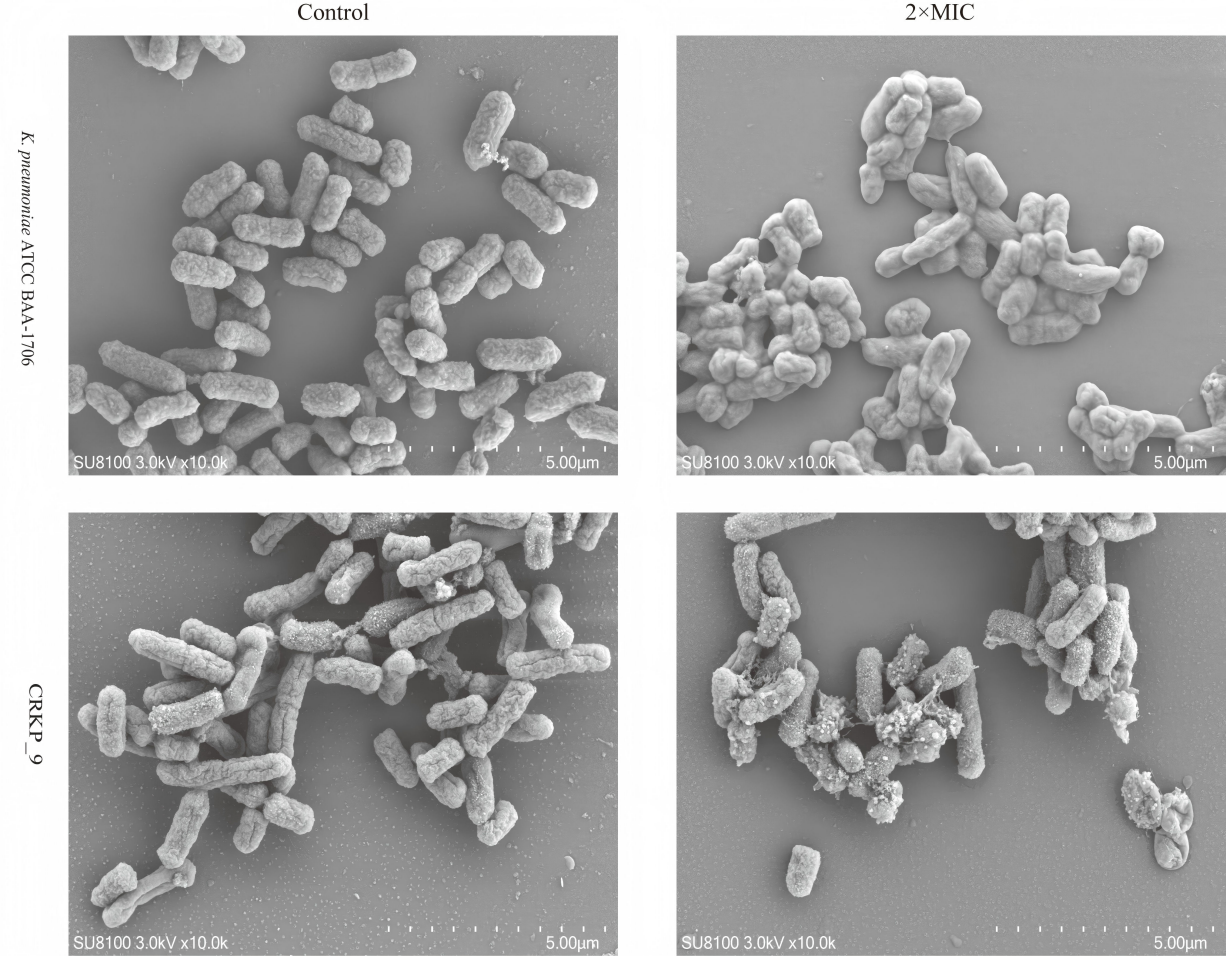
Morphological changes of CEC-TY1 after treatment of *K. pneumoniae* ATCC BAA-1706 and CRKP_9 were observed under scanning electrical environment, Bacterial solution concentration, 1 × 10^8^ CFU/mL.

To observe the antibacterial mechanism of CEC-TY1 against *K. pneumoniae* ATCC BAA-1706 and CRKP_9 in a more intuitive way, the bacteria were photographed using SEM before and after being treated with CEC-TY1. As shown in Fig. 3, the bacterial cells in the control group had intact cell membranes with smooth and full surfaces and clear edges. After being treated with 2 × MIC of CEC-TY1, the bacteria showed obvious wrinkling, surface rupture, and exudation of contents, which was consistent with the results of PI fluorescent staining. This indicated that the cell membrane structure of the bacteria was destroyed after being treated with CEC-TY1, leading to the death of the bacteria.

### Determination of bacterial LD_50_

According to the pre-experiment, LD_0_ = 9 × 10^7^ CFU/mL and LD_100_ = 2 × 10^9^ CFU/mL for the standard strain of *K. pneumoniae* ATCC BAA-1706, and LD_0_ = 5 × 10^7^ CFU/mL and LD_100_ = 9 × 10^8^ CFU/mL for the clinical strain of CRKP_9. The survival of mice infected with bacteria by intraperitoneal injection at different challenge dose levels is shown in Tables 1 and 2.

**TABLE 1:** Survival of mice after infection with different concentrations of *K. pneumoniae* ATCC BAA-1706

Groups	Bacterial concentration (CFU/mL)	Total quantity	Number of deaths	Mortality rate (%)
1	9.5 × 10^8^	6	5	83.33
2	6.0 × 10^8^	6	4	66.67
3	4.5 × 10^8^	6	3	50.00
4	2.0 × 10^8^	6	2	33.33
5	1.0 × 10^8^	6	1	16.67

**TABLE 2:** Survival rate of CRKP_9-infected mice^*a*^

Groups	Bacterial concentration (CFU/mL)	Total quantity	Number of deaths	Mortality rate (%)
6	6.0 × 10^8^	6	5	83.33
7	1.0 × 10^8^	6	4	66.67
8	9.0 × 10^7^	6	4	66.67
9	8.0 × 10^7^	6	2	33.33
10	7.5 × 10^7^	6	2	33.33

^*a*^ Mortality rate = (number of deaths / number of animals) × 100%.

The LD_50_ values of *K. pneumoniae* ATCC BAA-1706 and CRKP_9 were 3.5 × 10^8^ CFU/mL and 0.9 × 10^8^ CFU/mL; these concentrations are suitable for the establishment of the mouse abdominal infection model.

### Cytotoxicity analysis of CEC-TY1

**Fig 4:**
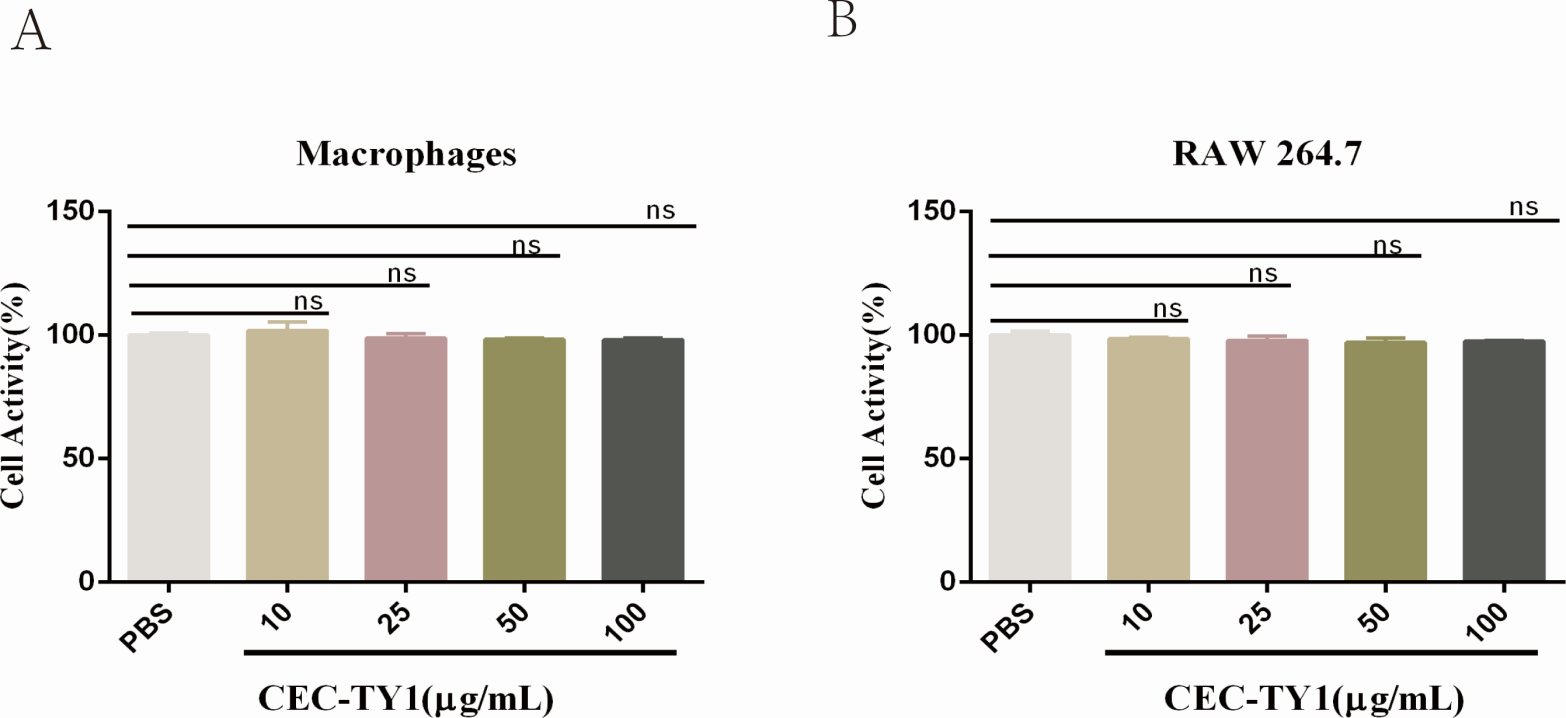
Cytotoxicity of CEC-TY1. The PBS group was the blank group, and the absorbance at 450 nm was determined; (**A**) mouse peritoneal macrophages; (**B**) RAW 264.7; ns, not statistically significant.

RAW 264.7 and MPMs were used evaluate the toxicity of antimicrobial peptides against mammalian cells. As shown in Fig. 4, the cell viability of RAW 264.7 and MPMs treated with CEC-TY1 was not significantly affected even when the concentration reached 100 µg/mL. This initial result indicates that CEC-TY1 has low toxicity *in vitro*, laying a foundation for the assessment of its safety and efficacy.

### Analysis of hemolytic activity of erythrocytes by CEC-TY1

**Fig 5:**
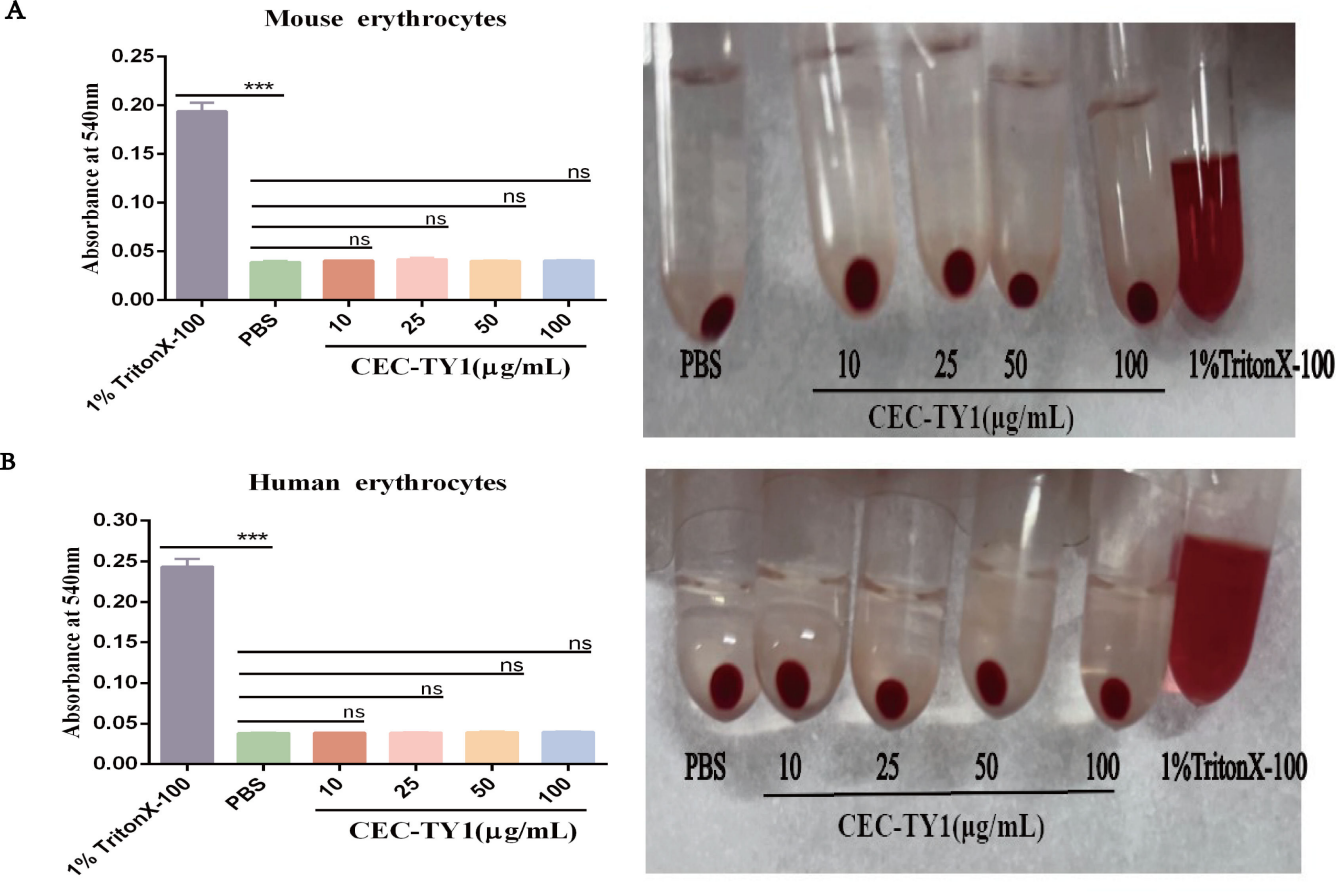
Erythrocyte hemolytic activity of CEC-TY1. PBS group was used as a blank group, 1% Triton X-100 was used as a positive control, and absorbance at 540 nm was measured; (**A**) mouse peripheral blood erythrocytes; (**B**) adult peripheral blood erythrocytes; ns, not statistically significant; ***P < 0.001.

Despite the strong antibacterial activity of AMPs, a large part of them also has a high hemolytic activity, which is an important factor hindering their clinical application. In order to verify the hemolytic activity of CEC-TY1, different concentrations of CEC-TY1 treatment groups were set up, and the experimental results are shown in Fig. 5. When compared with the PBS group (blank control group), no hemolysis was observed in red blood cells even when the dose reached 100 µg/mL, providing justification for the subsequent use of CEC-TY1 in *in vivo* experiments.

### CEC-TY1 can effectively protect mice from CRKP infection

**Fig 6:**
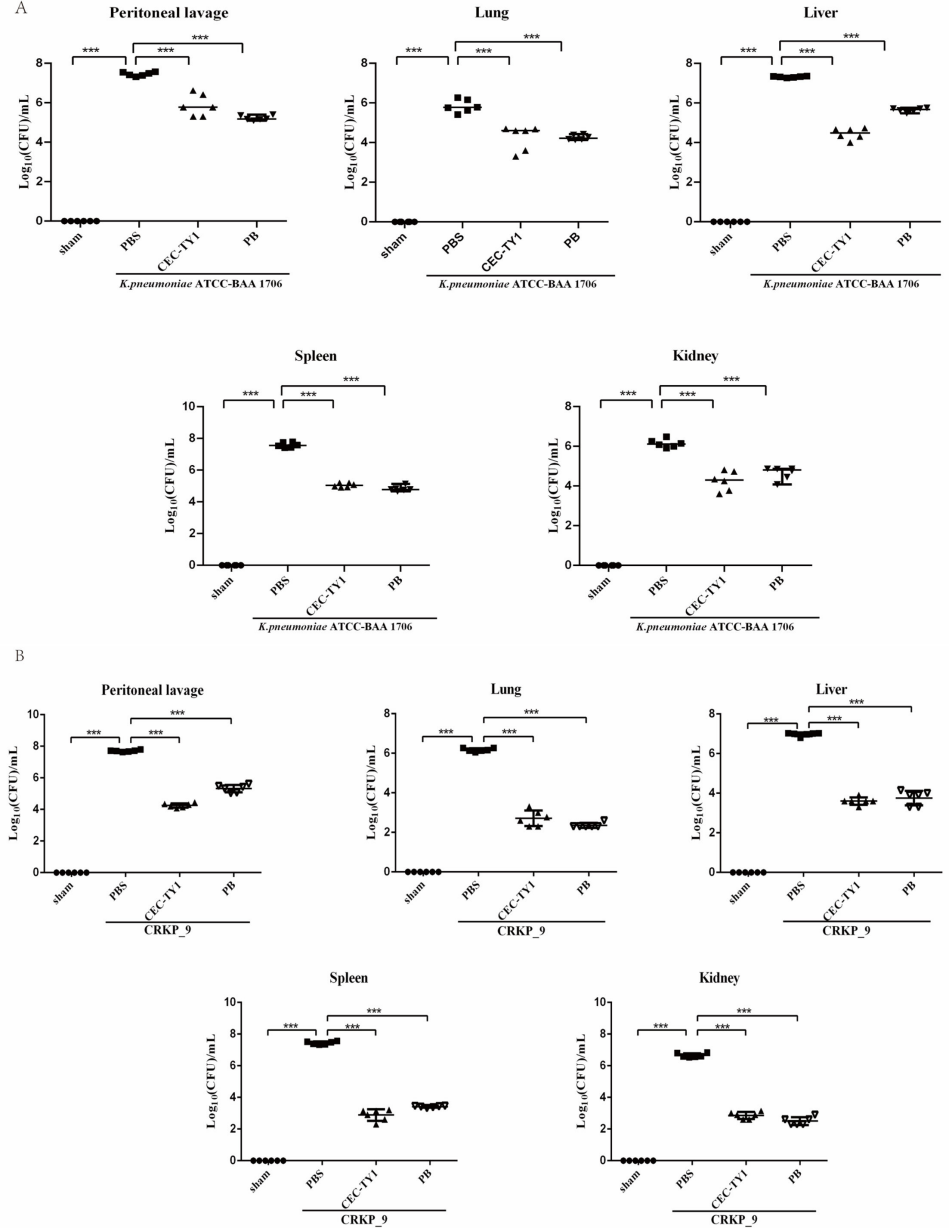
Intraperitoneal injection of CEC-TY1 2 h after infection reduces bacterial levels in the abdominal lavage fluid and vital tissue organs. PBS was the control group, CEC-TY1 was the experimental group, and PB was the positive control group.(**A**) Intraperitoneal injection of *K. pneumoniae* ATCC BAA-1706. (**B**) Intraperitoneal injection of CRKP_9. Scattered dots in the graph represent mice (six per group), ***P < 0.001.

CEC-TY1 reduced the bacterial load in the mouse intraperitoneal infection model. As previously mentioned, CEC-TY1 has strong antibacterial and anti-inflammatory effects, showing rapid and effective bactericidal ability against CRKP. Since CRKP often causes intraperitoneal infection, we tested the protective effect of CEC-TY1 *in vivo* by establishing a mouse model of intraperitoneal CRKP infection. First, the bacterial load of the mice after infection was measured. As shown in Fig. 6A and B, after intraperitoneal injection of CRKP in mice for 24 h, bacteria could be seen colonizing various organs of mice. After treatment with 10 mg/kg CEC-TY1, bacterial load in peritoneal lavage fluid and organs was significantly reduced compared with that in the PBS treatment group, the magnitude of the bacterial load reduction caused by CEC-TY1 was a least as large or larger than that of the polymyxin B positive control, indicating that CEC-TY1 has significant therapeutic potential for CRKP infection.

### CEC-TY1 can reduce intraperitoneal infection and inflammation caused by CRKP

**Fig 7:**
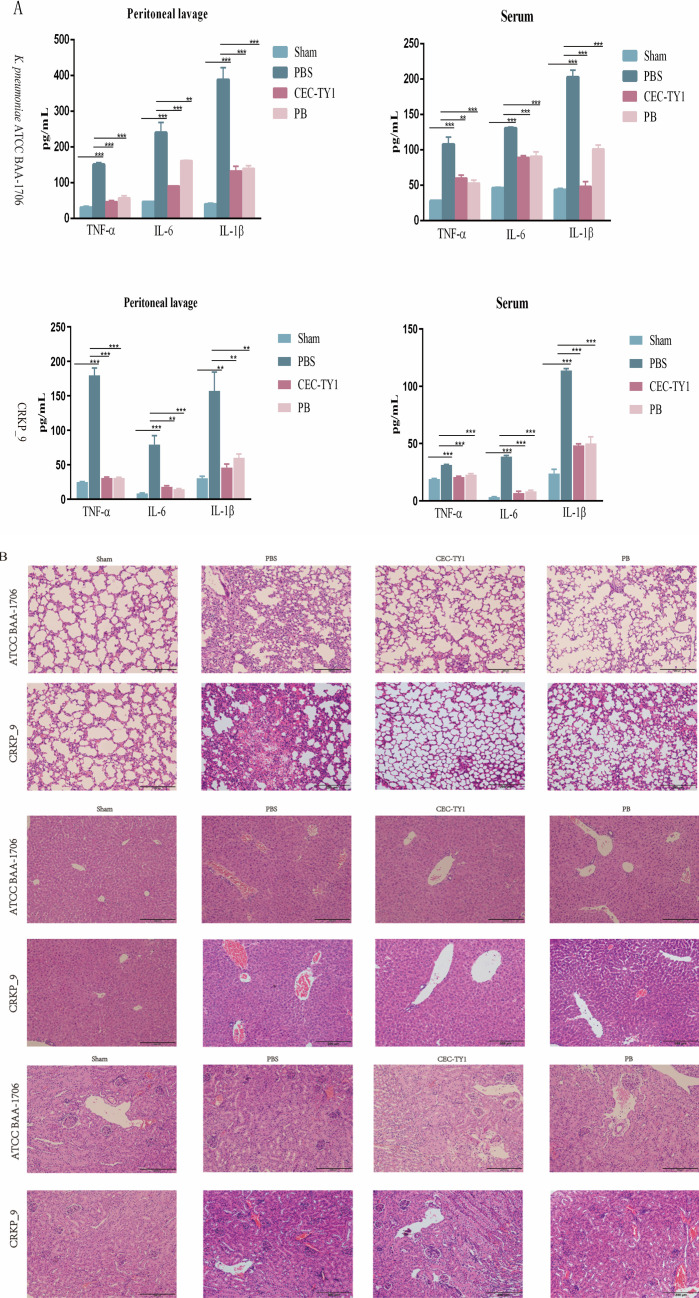
Intraperitoneal injection of CEC-TY1 can reduce CRKP-induced inflammation in vital tissues and organs (scale bar = 200 µm). (**A**) CEC-TY1 inhibits cytokine release in CRKP-induced mouse peritonitis models. (**B**) Tissue Hematoxylin and eosin staining. **P < 0.01, and ***P < 0.001.

We investigated the inflammatory response of bacteria-infected mice after treatment with CEC-TY1 to further explore its therapeutic potential. Compared with the sham group, CRKP infection significantly induced the production of IL-6, TNF-α, and IL-1β in the serum and peritoneal lavage fluid of mice, but significantly reduced the content of these cytokines after treatment with CEC-TY1, as shown in Fig. 7A. At the same time, the lungs, liver, and kidneys of mice infected with CRKP showed obvious congestion, edema, and a large number of inflammatory cell infiltration, but the organ damage was significantly reduced after treatment with CEC-TY1, as shown in Fig. 7B.

### CEC-TY1 increased the survival rate of CRKP-infected mice

**Fig 8:**
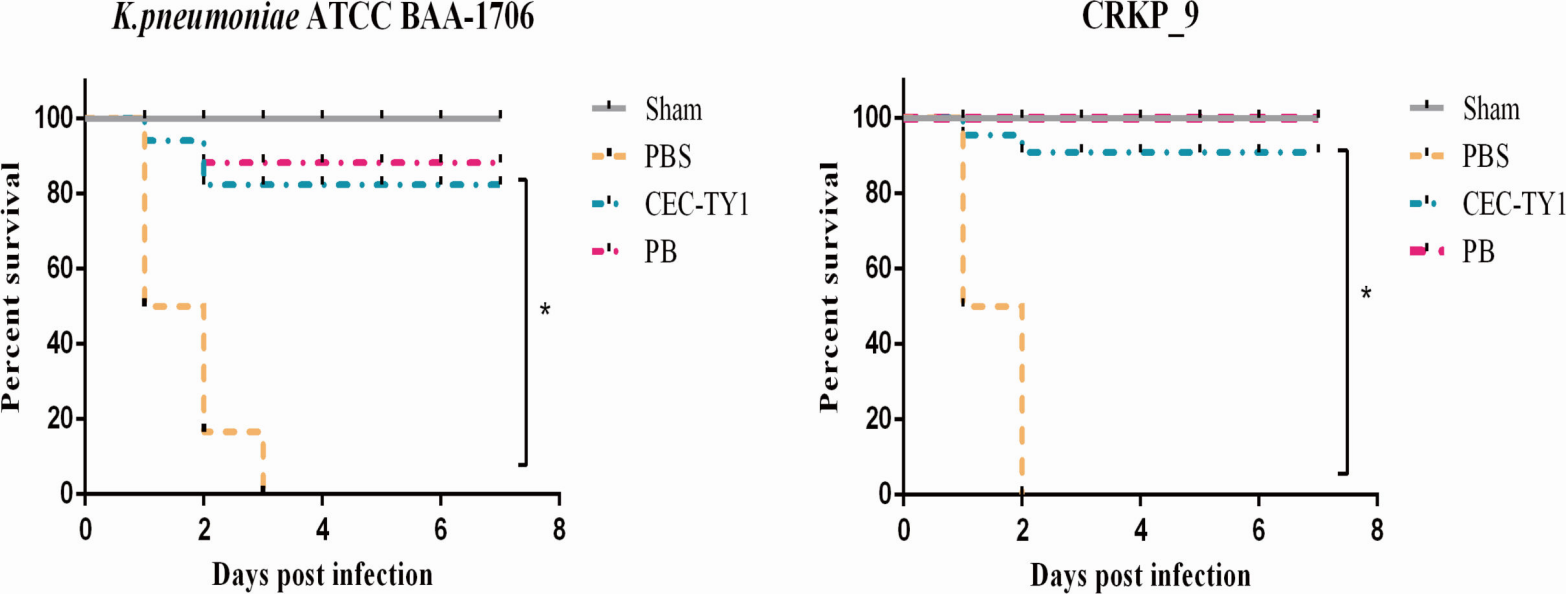
Intraperitoneal injection of CEC-TY1 after *K. pneumoniae* ATCC-BAA-1706 and CRKP_9 infections improves the survival rate of mice after bacterial infections. *K. pneumoniae* ATCC-BAA-1706 and CRKP_9 were used to infect mice for 2 h, and the survival rate of mice in each group was recorded by intraperitoneal injection of PBS (control), CEC-TY1 (10 mg/kg), and PB (10 mg/kg), and observed for 7 days. The survival rate of mice in each group was recorded, *P < 0.05.

Finally, we recorded the mortality rate of mice for 7 days. As shown in Fig. 8, the survival rate of mice treated with intraperitoneal injection of CEC-TY1 after CRKP infection was significantly improved, which is consistent with the effect of CEC-TY1 on bacterial colonization and inflammatory response. These results indicate that CEC-TY1 has good therapeutic effects in killing bacteria and inhibiting inflammatory responses caused by bacterial infection, similar to the results after PB treatment.

## DISCUSSION

AMPs have evolved for billions of years and are the first line of defense against pathogenic microorganisms in most animals. They have potential utility as an alternative to traditional antibiotics, and increasing attention is directed toward the activities of AMPs, especially their antibacterial activities (34, 35). In this study, the antibacterial activity of CEC-TY1 was verified *in vitro*. The MIC and MBC of CEC-TY1 against multiple strains of bacteria were determined experimentally, and the antibiotic PB was used as a positive control when the lowest inhibitory concentration of CEC-TY1 was determined. According to the data in Table 3, CEC-TY1 has strong antibacterial activity against gram-negative bacteria, with the lowest inhibitory concentration of 2 µg/mL–4 µg/mL, placing it in the strong antibacterial range. Similarly, a thioredoxin-conjugated recombinant form of another peptide derived from the same insect, Trx-stomoxynZH1 (36), was also better against gram-negative bacteria (15 µg/mL–30 µg/mL) than against gram-positive bacteria (27 µg/mL–54 µg/mL). These data also confirm the excellent potential efficacy of CEC-TY1 as an antibacterial compound. Furthermore, in a bactericidal kinetics experiment, CEC-TY1 exerted a rapid killing effect on *K. pneumoniae* ATCC BAA-1706 and CRKP.

**TABLE 3:** Antibacterial activity of CEC-TY1 against different gram-positive and gram-negative bacteria^*a*^

	Antibacterial drugs		
Bacterial strain	PB (MIC)	CEC-TY1 (MIC)	CEC-TY1 (MBC)
Gram-positive bacteria			
*S. aureus* ATCC 29213	–^*b*^	>64	–
*E. faecalis* ATCC 29212	–	>64	–
Gram-negative bacteria			
*K. pneumoniae* ATCC BAA-1706	2	2	4
CRKP_1	2	2	8
CRKP_5	2	2	8
CRKP_9	2	2	8
CRKP_11	2	2	8
CRKP_13	2	4	8
*E. coli* ATCC 25922	1	2	2
*P. aeruginosa* ATCC 27853	1	4	8

^*a*^ The data in this table uses uniform units of μg/mL.

^*b*^ –, item has not been tested.

AMPs kill bacteria predominantly by disrupting the structure of their cell membranes or by binding to multiple targets within the cell (such as DNA, RNA, or proteins) to restrict the normal metabolic processes of the bacteria (34, 37–39). These rapid-killing and membrane-damaging mechanisms do not target specific molecules or pathways, so bacterial resistance to AMPs cannot develop readily. Therefore, AMPs have broad potential applications in the treatment of drug-resistant bacterial infections (40). We detected the destruction of the bacterial structure by CEC-TY1 with SEM and an analysis of PI fluorescence intensity. SEM revealed changes in the bacterial morphology after treatment with CEC-TY1, confirming that CEC-TY1 destroys the integrity of the bacterial structure and causes the seepage of bacterial contents, resulting in bacterial death. Fluorescent staining with PI showed that the number of dead bacteria increased as the CEC-TY1 concentration increased, indicating that the bactericidal effect of CEC-TY1 involves the destruction of the cell structure of *K. pneumoniae* ATCC BAA-1706 and CRKP_9. These results are consistent with the previously reported antimicrobial mechanisms of short linear AMPs (SLAP)-S25 (41), ZY4 (42), and lipid peptide LP-20 (43).

The evaluation of the safety of an AMP is essential for its development as a new antibacterial drug. The cytotoxicity and hemolytic activities of AMPs are important determinants of their possible clinical application. CEC-TY1 caused no hemolysis or cytotoxicity while exerting its antibacterial activity, supporting its potential utility in systemic therapeutic applications. Therefore, we chose CEC-TY1 as a template for further studies *in vivo*. Although most AMPs have shown strong bactericidal effects in *in vitro* experiments, they usually lack effective antibacterial activity *in vivo* due to the complex environment in animals (44). Peritoneal infections cause serious functional damage to the organs in the abdomen, which usually manifests as the inflammation of a single organ or different forms of peritonitis. Infectious peritonitis is a local inflammatory process caused by bacteria and other pathogens, which is also called “abdominal sepsis” (45). Studies have shown that gram-negative bacilli most commonly cause abdominal infections and that resistance to carbapenems is increasing among gram-negative bacilli (46). In a mouse model of CRKP peritoneal infection, CEC-TY1 significantly reduced the bacterial load in the vital organs (such as the lungs, liver, kidney, and spleen) and peritoneal lavage fluid. Moreover, a survival analysis showed that CEC-TY1 treatment significantly improved the survival rate of the mice and effectively protected them from death caused by CRKP infection.

Bacterial infections often trigger the expression of inflammatory factors (47). If inflammation is not effectively controlled, it can also harm the host. Most AMPs in the cecropin family have anti-inflammatory activity. For instance, cecropin A not only effectively reduced the levels of proinflammatory factors TNF-α, IL-6, and IL-1β in the colon tissues of mice with ulcerative colitis but also reduced the *E. coli*-induced expression of IL-6, IL-8, and TNF-α mRNAs in porcine intestinal epithelial cells (IPEC-J2) (48, 49). *Musca domestica* cecropin effectively reduced the levels of IL-6 and other factors in the sera of mice after bacterial infection (50), and AeaeCec5 exerted a strong anti-inflammatory protective effect against gram-negative bacterial infection by inhibiting the expression of TNF-α, IL-1β, and IL-6 (51). In the present study, we have shown that CEC-TY1 also effectively controlled the CRKP-induced levels of TNF-α, IL-6, and IL-1β expressed by proinflammatory cells in the sera and peritoneal lavage fluid of mice and significantly prevented the damage to the lung, liver, and kidney usually observed after CRKP infection. Therefore, CEC-TY1 shows promise as a potential lead candidate for preventing infections caused by drug-resistant Gram-negative bacteria.

In summary, our study has shown that CEC-TY1 exerts a strong anti-inflammatory effect and a significant therapeutic effect on CRKP-induced intraperitoneal infection, significantly improving the survival rate in infected mice, reducing the bacterial loads in the intraperitoneal lavage fluid, kidney, liver, lung, and spleen, and reducing the degree of organ injury in the mice after infection. Our findings lay a foundation for the development of CEC-TY1 as a potential new antibacterial drug. However, due to time constraints, the risk of the development of resistance to CEC-TY1 by CRKP was not assessed in this study. Moreover, further investigation is required to verify the stability of CEC-TY1 *in vivo* and to determine whether combining it with antibiotics will improve its antibacterial effect.

### Conclusion

CEC-TY1 has significant antibacterial activity against gram-negative bacteria without hemolytic activity or cytotoxicity. It can kill bacteria quickly in a short time by destroying bacterial structure. At the same time, CEC-TY1 has an *in vivo* protective effect on mouse abdominal infection caused by CRKP and inhibits the invasion of major organs by bacteria through direct bactericidal activity. At the same time, inhibiting the production of proinflammatory cytokines can further alleviate the inflammatory response of mice infected in the abdominal cavity, reduce organ damage, and improve the survival rate of mice infected in the abdominal cavity. This study provides the first preclinical “proof-of-principle” for the antimicrobial CEC-TY1 class of peptides as a potential treatment for acute CRKP infections. Further studies will be required to assess its suitability for clinical development, such as structure-activity relationship profiling to optimize pharmacokinetics, bioavailability, stability, and other drug properties.
